# Risk of Rheumatoid Arthritis in Patients with Type 2 Diabetes: A Nationwide Population-Based Case-Control Study

**DOI:** 10.1371/journal.pone.0101528

**Published:** 2014-07-02

**Authors:** Ming-Chi Lu, Shih-Tang Yan, Wen-Yao Yin, Malcolm Koo, Ning-Sheng Lai

**Affiliations:** 1 Division of Allergy, Immunology and Rheumatology, Dalin Tzu Chi Hospital, Buddhist Tzu Chi Medical Foundation, Chiayi, Taiwan; 2 School of Medicine, Tzu Chi University, Hualien, Taiwan; 3 Division of Endocrinology and Metabolism, Dalin Tzu Chi Hospital, Buddhist Tzu Chi Medical Foundation, Chiayi, Taiwan; 4 Division of General Surgery, Dalin Tzu Chi Hospital, Buddhist Tzu Chi Medical Foundation, Chiayi, Taiwan; 5 Department of Medical Research, Dalin Tzu Chi Hospital, Buddhist Tzu Chi Medical Foundation, Chiayi, Taiwan; 6 Dalla Lana School of Public Health, University of Toronto, Ontario, Canada; University of Michigan Medical School, United States of America

## Abstract

**Objective:**

Type 2 diabetes is associated with chronic, low-grade inflammation and could potentially trigger the progression of other, more prominent inflammatory diseases such as rheumatoid arthritis (RA). Therefore, we aimed to investigate the risk of incident RA in Taiwanese patients with type 2 diabetes using a population-based health claims database.

**Methods:**

This nationwide, population-based, case-control study used administrative data to identify 1,416 patients with RA (age ≥20 years) as cases and 7,080 controls that were frequency-matched for sex, 10-year age group, and year of catastrophic illness certificate application date (index year). All subjects were retrospectively traced back, up to 13 years prior to the index year, for their first diagnosis of type 2 diabetes. Logistic regression analysis was conducted to quantify the association between incident RA and type 2 diabetes.

**Results:**

The odds of developing RA were significantly higher in female (odds ratio [OR] 1.46, 95% confidence interval [95% CI] 1.24–1.72) but not in male (OR 1.00, 95% CI 0.72–1.37) patients who had previously diagnosed with type 2 diabetes. Subgroup analysis indicated that the odds of developing RA were more prominent in younger females (20 to 44 years of age) with type 2 diabetes. In addition, the odds of developing RA in female patients with type 2 diabetes were higher in those with a shorter time interval between the diagnosis of type 2 diabetes and RA.

**Conclusions:**

This large nationwide, population-based, case-control study showed an elevated risk of RA in female Taiwanese patients with type 2 diabetes. Our findings were consistent with the hypothesis that chronic low-grade inflammation in type 2 diabetes may elicit the development of RA in genetically susceptible individuals.

## Introduction

The prevalence of type 2 diabetes has rapidly increased in many Asian populations, including the Taiwanese, thought to be the result of a combination of a sedentary lifestyle and unhealthy dietary habits. In Taiwan, the crude incidence of type 2 diabetes was 8 per 1,000 with a prevalence of 8.3% in 2007 [1]. Type 2 diabetes is characterized by pancreatic β cell dysfunction and insulin resistance. Obesity can trigger chronic low-grade inflammation and the resulting inflammatory mediators are detrimental to β cell function [2], [3]. Modulating inflammatory reactions in patients with type 2 diabetes with the use of salsalate has been shown to improve glycemic control [6], strongly suggesting that inflammatory pathways are involved in the metabolic abnormalities of type 2 diabetes.

Rheumatoid arthritis (RA) is a chronic and disabling disease characterized by persistent synovitis, systemic inflammation, and the presence of autoantibodies [7]. The annual incidence of RA in Taiwan was 15.8 cases per 100,000 population with a female to male ratio of 4 to 1 [8]. Inflammatory mediators such as C-reactive protein (CRP), interleukin (IL)-6, and tumor necrosis factor (TNF)-*α* are frequently elevated in patients with type 2 diabetes [4], [5] as well as in the sera of patients many years before the clinical onset of RA [9], suggesting a critical role in the immunopathogenesis of this disease. This observation also suggests that low-grade inflammation should have already existed in patients with RA during the preclinical phase. In addition, smoking causes chronic lung inflammation and could subsequently lead to the production of autoantibodies, resulting in the development of RA among genetically susceptible individuals [10]. Therefore, it is plausible that chronic low-grade inflammation observed in patients with type 2 diabetes could also contribute to the development of RA in these patients. Several studies show the risk of diabetes is higher in patients with RA, but less is known about RA risk in established type 2 diabetes [11], [12]. Therefore, the aim of this case-control study was to explore the risk of incident RA in patients with type 2 diabetes using a nationwide health claims database. To our knowledge, this is the first study to investigate the risk of incident RA among patients with type 2 diabetes using nationwide, population-based data.

## Materials and Methods

### Data sources and study subjects

This study used a nationwide, population-based, case-control study design based on the data available from the National Health Insurance Research Database (NHIRD) in Taiwan [13].

The study protocol was reviewed and approved by the institutional review board of the Dalin Tzu Chi Hospital, Buddhist Tzu Chi Medical Foundation, Taiwan (No. 10004021). Since the NHIRD datafiles contain only de-identified secondary data, the need for informed consent from individual subjects was waived.

Using the 1997–2010 catastrophic illness datafile, a part of the NHIRD, cases were defined as new and successful applicants for the certificate of catastrophic illness with RA (International Classification of Diseases, ninth revision, clinical modification, ICD9-CM code 714.0). In Taiwan, patients with RA can apply for catastrophic illness certificates from the Bureau of National Health Insurance to become exempt from copayments for healthcare costs related to RA. The certificate is issued to patients only after their medical records, laboratory data, and imaging results have been reviewed by at least two rheumatologists. Because RA and type 2 diabetes occur primarily in adults, patients under 20 years of age were excluded from the study. The index date was defined as the date of first application of catastrophic illness certificate in the cases. A total of 1,416 patients with RA were included in the analyses.

The controls were selected from a random sample of the ambulatory care datafile of the 2000 Longitudinal Health Insurance Database (LHID2000) within the same inclusion period (January 1, 1997 to December 31, 2010) as the cases. The LHID2000 contained the claim data and registration files of both ambulatory and inpatient care expenditures for one million individuals randomly sampled from the 2000 Registry for Beneficiaries of the NHIRD. Any individual who was once a beneficiary of the National Health Insurance program during the period of 1996 to 2000 would be included in the registry. There are approximately 23,720,000 individuals in this registry, which represents more than 99% of the population in Taiwan. The National Health Research Institute confirmed that there were no significant differences in the age and sex between the LHID2000 and all beneficiaries.

Individuals identified as cases were excluded from the selection. Five controls per case were selected based on frequency matching for sex, 10-year age interval, and index year. Therefore, a total of 7,080 controls were included in the statistical analyses. The index year was defined as the same year of the index date of the matched case. The index date for each of the control subjects was assigned by using the date of a randomly selected ambulatory visit for a given index year.

Both the cases and controls were linked to the LHID2000 to obtain their ambulatory care visit data from 1997 to 2010. Patients were identified as having type 2 diabetes if they had at least three instances of ambulatory claims with a diagnosis of A-code A181 or ICD9-CM code 250.xx, except type 1 diabetes (ICD9-CM code 250.x1 or 250.x3). Patients with type 1 diabetes were excluded from this study because the disease is associated with an increased risk of several autoimmune diseases [14]. In addition, patients diagnosed with type 2 diabetes after the index date were excluded from the analyses. Therefore, only incident RA occurring after a diagnosis of type 2 diabetes were included in the final analyses.

### Statistical analysis

Sex and age groups between cases and controls were compared with Chi-square test and t-test, as appropriate. Logistic regression was used to determine the odds ratio (OR) and 95% confidence interval (95% CI) of the risk of RA development with type 2 diabetes. A two-tailed *P* value of <0.05 was considered statistically significant. All statistical analyses were conducted using IBM SPSS Statistics software package, version 21.0 (IBM Corp, Armonk, NY, USA).

## Results

We identified 1,416 patients newly diagnosed with RA with a certificate of catastrophic illness as cases, and 7,080 non-RA controls frequency-matched for sex, 10-year age interval, and index year. Table 1 shows the distribution of age and time interval between the first diagnosis of type 2 diabetes and index date in the cases and controls, stratified by sex. The mean age was 53.9 years for cases and the female to male ratio was 3.4∶1. Since we frequency matched the cases and controls, there were no significant differences in their age distribution, with or without stratification by sex. However, there were significantly more patients with a longer time interval between the first diagnosis of type 2 diabetes and the index date among controls than among the cases (*P* = 0.003); this difference was observed in females (*P* = 0.024) but not in males (*P* = 0.118).

**Table 1:** Characteristics of patients with rheumatoid arthritis and frequency-matched controls (N = 8,496).

Variable		Total			Males			Females		
		n (%)		*P* value	n (%)		*P* value	n (%)		*P* value
		Cases	Controls		Cases	Controls		Cases	Controls	
		1,416 (16.7)	7,080 (83.3)		320 (16.7)	1,600 (83.3)		1,096 (16.7)	5,480 (83.3)	
Age (years)				>0.999			>0.999			>0.999
	20–29	68 (4.8)	340 (4.8)		13 (4.1)	65 (4.1)		55 (5.0)	275 (5.0)	
	30–39	161 (11.4)	805 (11.4)		22 (6.9)	110 (6.9)		139 (12.7)	695 (12.7)	
	40–49	310 (21.9)	1,550 (21.9)		72 (22.5)	360 (22.5)		238 (21.7)	1,190 (21.7)	
	50–59	398 (28.1)	1,990 (28.1)		85 (26.6)	425 (26.6)		313 (28.6)	1,565 (28.6)	
	60–69	267 (18.9)	1,335 (18.9)		53 (16.6)	265 (16.6)		214 (19.5)	1,070 (19.5)	
	70–79	166 (11.7)	830 (11.7)		60 (18.8)	300 (18.8)		106 (9.7)	530 (9.7)	
	80–89	42 (3.0)	210 (3.0)		14 (4.4)	70 (4.4)		28 (2.6)	140 (2.6)	
	90–99	4 (0.3)	20 (0.3)		1 (0.3)	5 (0.3)		3 (0.3)	15 (0.3)	
	mean (SD)	53.9 (14.1)	53.7 (14.5)	0.749	56.7 (14.4)	56.4 (15.0)	0.765	53.1 (13.9)	53.0 (14.2)	0.842
Interval between T2D diagnosis and index date (years)				0.003			0.118			0.024
	<4	72 (25.9)	196 (17.9)		16 (29.6)	51 (18.8)		56 (25.0)	145 (17.6)	
	4–<7	66 (23.7)	235 (21.5)		15 (27.8)	64 (23.6)		51 (22.8)	171 (20.8)	
	7–<11	76 (27.3)	308 (28.2)		14 (25.9)	74 (27.3)		62 (27.7)	234 (28.5)	
	≥11	64 (23.0)	354 (32.4)		9 (16.7)	82 (30.3)		55 (24.6)	272 (33.1)	

% are column percentages except in the header rows where they are row percentages.

T2D, type 2 diabetes.

Overall, the prevalence of type 2 diabetes was higher among RA cases (19.6%) compared with controls (15.4%) (Table 2). The odds of developing RA were significantly higher among patients who had previously diagnosed with type 2 diabetes (OR 1.34, 95% CI 1.16–1.55). When the data were analyzed after separating by sex, only female patients showed a significant association between RA and type 2 diabetes (OR 1.46, 95% CI 1.24–1.72). Therefore, we only included females in the subsequent analyses with stratification by age group (Table 3) and the time interval between the first diagnosis of type 2 diabetes and the index date (Table 4).

**Table 2:** Association between type 2 diabetes and incident rheumatoid arthritis in male and female patients (N = 8,496).

	Total, n (row %) 8,496 (100)		Sex, n (row %)			
			Male		Female	
			1,920 (22.6)		6,576 (77.4)	
	Cases	Controls	Cases	Controls	Cases	Controls
	1,416 (16.7)	7,080 (83.3)	320 (16.7)	1,600 (83.3)	1,096 (16.7)	5,480 (83.3)
T2D, n (column %)	278 (19.6)	1,093 (15.4)	54 (16.9)	271 (16.9)	224 (20.4)	822 (15.0)
Odds ratio (95% CI)	1.34 (1.16–1.55)	1.00	1.00 (0.72–1.37)	1.00	1.46 (1.24–1.72)	1.00
*P* value	<0.001		0.978		<0.001	

T2D, type 2 diabetes; 95% CI, 95% confidence interval.

**Table 3:** Association between type 2 diabetes and incident rheumatoid arthritis in female patients, stratified by age group (N = 6,576).

	Age group (years), n (row %)					
	20–44		45–64		≥65	
	1,836 (27.9)		3,282 (49.9)		1,458 (22.2)	
	Cases	Controls	Cases	Controls	Cases	Controls
	295 (16.1)	1,541 (83.9)	573 (17.5)	2,709 (82.5)	228 (15.6)	1,230 (84.4)
T2D, n (column %)	19 (6.4)	29 (1.9)	120 (20.9)	426 (15.7)	85 (37.3)	367 (29.8)
Odds ratio (95% CI)	3.59 (1.99–6.49)	1.00	1.42 (1.13–1.78)	1.00	1.40 (1.04–1.88)	1.00
*P* value	<0.001		0.002		0.026	

T2D, type 2 diabetes; 95% CI, 95% confidence interval.

**Table 4:** Association between type 2 diabetes and incident rheumatoid arthritis in female patients, stratified by the time interval between the first diagnosis of type 2 diabetes and the index date (N = 6,576).

	Time interval between the first diagnosis of type 2 diabetes mellitus and the index date (years), n (row %)							
	<4		4–<7		7–<11		≥11	
	1,763 (26.8)		1,501 (22.8)		1,594 (24.2)		1,718 (26.1)	
	Cases	Controls	Cases	Controls	Cases	Controls	Cases	Controls
	281 (15.9)	1,482 (84.1)	252 (16.8)	1,249 (83.2)	269 (16.9)	1,325 (83.1)	294 (17.1)	1,424 (82.9)
T2D, n (column %)	56 (19.9)	145 (9.8)	51 (20.2)	171 (13.7)	62 (23.0)	234 (17.7)	55 (18.7)	272 (19.1)
Odds ratio (95% CI)	2.30 (1.64–3.22)	1.00	1.60 (1.13–2.26)	1.00	1.40 (1.02–1.92)	1.00	0.98 (0.71–1.34)	1.00
*P* value	<0.001		0.008		0.039		0.876	

T2D, type 2 diabetes; 95% CI, 95% confidence interval.

Subgroup analyses with stratification by age group indicated that the odds of developing RA in patients with type 2 diabetes were highest (OR 3.59, 95% CI 1.99–6.49) for the youngest age group of 20 to 44 years. The magnitude of the association was statistically significant but smaller in the two older age groups. Furthermore, stratification by the time interval between the first diagnosis of type 2 diabetes and the index date in female patients showed an increased odds of developing RA within a shorter time interval. The association was strongest with an interval of <4 years (OR 2.30, 95% CI 1.64–3.22) compared to those with an interval of 4 to <7 years (OR 1.60, 95% CI 1.13–2.26) or an interval of 7 to 11 years (OR 1.40, 95% CI 1.02–1.92). No significant associations were observed in patients with an interval of ≥11 years (OR 0.98, 95% CI 0.71–1.34).

## Discussion

Previous studies based on healthcare utilization data have reported an increased risk of type 2 diabetes in patients with RA [11], [12]. In contrast, our study showed that female patients with type 2 diabetes were associated with a significantly increased risk for incident RA. The increased risk of type 2 diabetes in patients with RA was proposed to be due to the long-term use of steroids during RA treatment [15]. Nevertheless, a Canadian study using a population-based health insurance database demonstrated a similar risk of incident type 2 diabetes in patients with RA, with or without adjusting for the use of oral or topical glucocorticoids [12]. Conversely, decreased insulin sensitivity was reported in patients with RA upon long-term exposure to steroids [16]. Thus, the role of long-term steroid use among patients with RA in the development of type 2 diabetes still requires further investigation. Furthermore, lifestyle changes after diagnosis of RA [17] might also contribute to an increase in the risk of developing type 2 diabetes. One health insurance database study performed in the United Kingdom concluded that the observed association between patients with RA and incident type 2 diabetes could substantially explained by obesity and lifestyle factors [18].

Our findings also showed that the risks between type 2 diabetes and incident RA were more prominent in younger female patients and in those with a shorter interval between the diagnosis of type 2 diabetes and RA. In the youngest age group of 20 to 44 years, the risk of developing RA was 3.6 times higher in patients with type 2 diabetes compared with patients without type 2 diabetes. Since both RA and type 2 diabetes are primarily chronic diseases of an older population, a strong positive association in young female patients is of concern. It is possible that these patients are genetically susceptible for developing autoimmune diseases, which tends to manifest early in life [19].

Furthermore, the association between type 2 diabetes and incident RA was found to be strongest for patients with the shortest time interval (<4 years) between the diagnosis of type 2 diabetes and the index date. Conversely, patients whose RA appeared 11 years or more after the diagnosis of type 2 diabetes were not significantly associated with the presence of type 2 diabetes. The proximity in the timing between when these two conditions occurred is consistent with the notion that type 2 diabetes and RA are related.

The possible link between type 2 diabetes and incident RA could be explained by the inflammatory responses associated with both conditions. Serum levels of IL-6 and levels of CRP are significantly elevated in patients with RA [20]. Regarding type 2 diabetes, a meta-analysis of ten prospective studies with a total of 19,709 participants revealed a significant dose-response association of IL-6 levels with risk of type 2 diabetes (relative risk, [RR] 1.31, 95% CI 1.17–1.46). This same study, based on the results from 22 cohorts of 40,735 participants, also found that elevated CRP levels were significantly associated with increased risk of type 2 diabetes (RR 1.26, 95% CI 1.16–1.37) [21]. Furthermore, in patients with inflammatory polyarthritis, insulin resistance was closely associated with the presence of rheumatoid factor (RF) and anti-citrullinated protein antibody (ACPA) [22]. These findings suggest an important role for chronic systemic inflammation in the pathogenesis of both RA and type 2 diabetes.

The use of disease-modifying antirheumatic drugs (DMRADs) such as hydroxychloroquine or tumor necrosis factor inhibitor for lowering the inflammatory response in patients with RA has been shown to reduce the risk of developing diabetes [23], [24]. Altogether, these findings suggest that the pathogenesis of type 2 diabetes and RA could be linked by a similar chronic inflammatory response profile. Nevertheless, it should be noted that there is currently no evidence demonstrating that both conditions have shared genetic mechanisms [25].

The data used in this study were based on health claims records and thus could minimize the presence of recall bias as a result of collecting exposure information prior to disease onset in typical case-control studies. Still, there were several drawbacks in our study. First, due to the lack of detailed clinical data in the NHIRD, no information in regards to radiographic reports, biochemical and serological data (such as inflammatory markers, ACPA, and RF), or lifestyle factors such as smoking was available. Although smoking is an important risk factor for RA [26], the prevalence of female smokers is under 5%, and even lower (1.8%) among older women in Taiwan [27]. Thus, the increased risk of RA in female patients with type 2 diabetes should not be affected by smoking. On the other hand, prevalence of smoking was high (45.7%) in males and could confound the association between type 2 diabetes and RA, possibly leading to a lack of association between the two diseases in male patients. Since both the humoral and cell-mediated immune responses are more active in females, it is plausible that the disease expression is related to sexual dimorphism in the immune response [28]. This might also explain a significant association was only found in females, not male patients as well.

Second, although mobility and physical activity could be adversely affected by joint symptoms in individuals many years prior to the definitive diagnosis of RA [29], which in turn can increase the risk of type 2 diabetes, the joint symptoms before the clinical onset of RA are generally mild [30]. Therefore, the effect of immobility and physical inactivity on the development of type 2 diabetes should be minimal.

Third, it is possible that the association between RA and type 2 diabetes might be partially explained by the confounding effect of obesity. Nevertheless, although obesity is a well-established risk factor for type 2 diabetes [31], previous research on the association between body mass index and RA generated inconsistent results. Early studies had indicated a moderate increase in the risk of RA with obesity [32] but newer studies generally reported that obesity was not a predisposing factor for RA [33]. In addition, a comparison study using nationally representative survey data indicated that the prevalence of obesity was lower in Taiwan (7.2%) compared with the United States (33.7%) and England (32.1%) [34]. Furthermore, no significant differences in waist circumference were found between 86 Taiwanese patients with RA and 172 age- and sex-matched population controls [35]. Therefore, it might be premature to attribute the significant association between type 2 diabetes and incident RA to the confounding effect of obesity without further investigation.

In conclusion, this large nationwide, population-based, case-control study suggested that female patients with type 2 diabetes had an increased risk of developing RA. Although our findings are consistent with the hypothesis that chronic inflammation seen in patients with type 2 diabetes could contribute to the development of RA, further investigation with adjustments for possible confounders is needed before conclusions can be drawn. Nevertheless, given the high and increasing prevalence of type 2 diabetes in Taiwanese and other Asian populations, it is important to understand the effects of type 2 diabetes on the development of RA, particularly in young females.

## References

[1] Lin CC, Li CI, Hsiao CY, Liu CS, Yang SY, et al (2013) Time trend analysis of the prevalence and incidence of diagnosed type 2 diabetes among adults in Taiwan from 2000 to 2007: a population-based study. BMC Public Health 13: 318. 23570503 10.1186/1471-2458-13-318 PMC3626657

[2] Cefalu WT (2009) Inflammation, insulin resistance, and type 2 diabetes: back to the future? Diabetes 58: 307–308. 19171748 10.2337/db08-1656 PMC2628602

[3] Kolb H, Mandrup-Poulsen T (2005) An immune origin of type 2 diabetes? Diabetologia 48: 1038–1050. 15864529 10.1007/s00125-005-1764-9

[4] Aroor AR, McKarns S, Demarco VG, Jia G, Sowers JR (2013) Maladaptive immune and inflammatory pathways lead to cardiovascular insulin resistance. Metabolism 62: 1543–1552. 23932846 10.1016/j.metabol.2013.07.001 PMC3809332

[5] Goldberg RB (2009) Cytokine and cytokine-like inflammation markers, endothelial dysfunction, and imbalanced coagulation in development of diabetes and its complications. J Clin Endocrinol Metab 94: 3171–3182. 19509100 10.1210/jc.2008-2534

[6] Goldfine AB, Fonseca V, Jablonski KA, Chen YD, Tipton L, et al (2013) Salicylate (salsalate) in patients with type 2 diabetes: a randomized trial. Ann Intern Med 159: 1–12. 23817699 10.7326/0003-4819-159-1-201307020-00003 PMC4128629

[7] Scott DL, Wolfe F, Huizinga TW (2010) Rheumatoid arthritis. Lancet 376: 1094–1108. 20870100 10.1016/S0140-6736(10)60826-4

[8] Kuo CF, Luo SF, See LC, Chou IJ, Chang HC, et al (2013) Rheumatoid arthritis prevalence, incidence, and mortality rates: a nationwide population study in Taiwan. Rheumatol Int 33: 355–360. 22451027 10.1007/s00296-012-2411-7

[9] van Steenbergen HW, Huizinga TW, van der Helm-van Mil AH (2013) The preclinical phase of rheumatoid arthritis: what is acknowledged and what needs to be assessed? Arthritis Rheum 65: 2219–2232. 23686440 10.1002/art.38013

[10] Baka Z, Buzás E, Nagy G (2009) Rheumatoid arthritis and smoking: putting the pieces together. Arthritis Res Ther 11: 238. 19678909 10.1186/ar2751 PMC2745780

[11] Su CC, Chen I, Young FN, Lian I (2013) Risk of diabetes in patients with rheumatoid arthritis: a 12-year retrospective cohort study. J Rheumatol 40: 1513–1518. 23818711 10.3899/jrheum.121259

[12] Solomon DH, Love TJ, Canning C, Schneeweiss S (2010) Risk of diabetes among patients with rheumatoid arthritis, psoriatic arthritis and psoriasis. Ann Rheum Dis 69: 2114–2117. 20584807 10.1136/ard.2009.125476 PMC2991521

[13] National Health Research Institute (2013) National Health Insurance Research Database, Taiwan. Available: http://nhird.nhri.org.tw/en/index.htm. Accessed 2014 May 23.

[14] Hemminki K, Li X, Sundquist J, Sundquist K (2009) Familial association between type 1 diabetes and other autoimmune and related diseases. Diabetologia 52: 1820–1828. 19543881 10.1007/s00125-009-1427-3

[15] Caldwell JR, Furst DE (1991) The efficacy and safety of low-dose corticosteroids for rheumatoid arthritis. Semin Arthritis Rheum 21: 1–11. 10.1016/0049-0172(91)90051-z 1948096

[16] Dessein PH, Joffe BI, Stanwix AE, Christian BF, Veller M (2004) Glucocorticoids and insulin sensitivity in rheumatoid arthritis. J Rheumatol 31: 867–874. 15124244

[17] Mancuso CA, Rincon M, Sayles W, Paget SA (2007) Comparison of energy expenditure from lifestyle physical activities between patients with rheumatoid arthritis and healthy controls. Arthritis Rheum 57: 672–678. 17471544 10.1002/art.22689

[18] Dubreuil M, Rho YH, Man A, Zhu Y, Zhang Y, et al (2013) Diabetes incidence in psoriatic arthritis, psoriasis and rheumatoid arthritis: a UK population-based cohort study. Rheumatology 53: 346–352. 24185762 10.1093/rheumatology/ket343 PMC3894671

[19] Kim EJ, Lee J, Ryu YS, Kim JM, Jeong YG, et al (2013) Shared epitope and radiologic progression are less prominent in elderly onset RA than young onset RA. Rheumatol Int 33: 2135–2140. 23443331 10.1007/s00296-013-2670-y

[20] Chung SJ, Kwon YJ, Park MC, Park YB, Lee SK (2011) The correlation between increased serum concentrations of interleukin-6 family cytokines and disease activity in rheumatoid arthritis patients. Yonsei Med J 52: 113–120. 21155043 10.3349/ymj.2011.52.1.113 PMC3017685

[21] Wang X, Bao W, Liu J, Ouyang YY, Wang D, et al (2013) Inflammatory markers and risk of type 2 diabetes: a systematic review and meta-analysis. Diabetes Care 36: 166–175. 23264288 10.2337/dc12-0702 PMC3526249

[22] Mirjafari H, Farragher TM, Verstappen SM, Yates A, Bunn D, et al (2011) Seropositivity is associated with insulin resistance in patients with early inflammatory polyarthritis: results from the Norfolk Arthritis Register (NOAR): an observational study. Arthritis Res Ther 13: R159. 21959060 10.1186/ar3476 PMC3308090

[23] Solomon DH, Massarotti E, Garg R, Liu J, Canning C, et al (2011) Association between disease-modifying antirheumatic drugs and diabetes risk in patients with rheumatoid arthritis and psoriasis. JAMA 305: 2525–2531. 21693740 10.1001/jama.2011.878

[24] Wasko MC, Hubert HB, Lingala VB, Elliott JR, Luggen ME, et al (2007) Hydroxychloroquine and risk of diabetes in patients with rheumatoid arthritis. JAMA 298: 187–193. 17622600 10.1001/jama.298.2.187

[25] Wellcome Trust Case Control Consortium (2007) Genome-wide association study of 14,000 cases of seven common diseases and 3,000 shared controls. Nature 447: 661–678. 17554300 10.1038/nature05911 PMC2719288

[26] Stolt P, Bengtsson C, Nordmark B, Lindblad S, Lundberg I, et al (2003) Quantification of the influence of cigarette smoking on rheumatoid arthritis: results from a population based case-control study, using incident cases. Ann Rheum Dis 62: 835–841. 12922955 10.1136/ard.62.9.835 PMC1754669

[27] Tsai YW, Tsai TI, Yang CL, Kuo KN (2008) Gender differences in smoking behaviors in an Asian population. J Womens Health 17: 971–978. 10.1089/jwh.2007.0621 PMC2942753 18681817

[28] Bouman A, Heineman MJ, Faas MM (2005) Sex hormones and the immune response in humans. Hum Reprod Update 11: 411–423. 15817524 10.1093/humupd/dmi008

[29] Lai NS, Tsai TY, Li CY, Koo M, Yu CL, et al (2014) Increased frequency and costs of ambulatory medical care utilization prior to the diagnosis of rheumatoid arthritis: A national population-based study. Arthritis Care Res (Hoboken) 66: 371–378. 23983071 10.1002/acr.22146

[30] van de Sande MG, de Hair MJ, van der Leij C, Klarenbeek PL, Bos WH, et al (2011) Different stages of rheumatoid arthritis: features of the synovium in the preclinical phase. Ann Rheum Dis 70: 772–777. 21177292 10.1136/ard.2010.139527

[31] Abdullah A, Peeters A, de Courten M, Stoelwinder J (2010) The magnitude of association between overweight and obesity and the risk of diabetes: a meta-analysis of prospective cohort studies. Diabetes Res Clin Pract 89: 309–319. 20493574 10.1016/j.diabres.2010.04.012

[32] Voigt LF, Koepsell TD, Nelson JL, Dugowson CE, Daling JR (1994) Smoking, obesity, alcohol consumption, and the risk of rheumatoid arthritis. Epidemiology 5: 525–532. 7986867

[33] Rodriguez LA, Tolosa LB, Ruigomez A, Johansson S, Wallander MA (2009) Rheumatoid arthritis in UK primary care: incidence and prior morbidity. Scand J Rheumatol 38: 173–177. 19117247 10.1080/03009740802448825

[34] Vasunilashorn S, Kim JK, Crimmins EM (2013) International differences in the links between obesity and physiological dysregulation: the United States, England, and Taiwan. J Obes 2013: 618056. 23781331 10.1155/2013/618056 PMC3679767

[35] Chen MF, Chen YM, Chen DY, Lan JL (2010) Metabolic syndrome in patients with rheumatoid arthritis: association of lifestyle with anthropometric measurements. Formosan J Rheumatol 24: 64–71.

